# Postoperative mortality in patients on chronic dialysis following elective surgery: A systematic review and meta-analysis

**DOI:** 10.1371/journal.pone.0234402

**Published:** 2020-06-26

**Authors:** Dharmenaan Palamuthusingam, Arun Nadarajah, Elaine M. Pascoe, Jonathan Craig, David W. Johnson, Carmel M. Hawley, Magid Fahim

**Affiliations:** 1 Metro South and Integrated Nephrology and Transplant Services, Logan Hospital, Meadowbrook, Queensland, Australia; 2 Faculty of Medicine, University of Queensland, St Lucia, Queensland, Australia; 3 School of Medicine, Griffith University, Mount Gravatt, Queensland, Australia; 4 Department of Surgery, Sunshine Coast University Hospital, Birtinya, Queensland, Australia; 5 Centre for Health Services Research, University of Queensland, St Lucia, Queensland, Australia; 6 College of Medicine and Public Health, Flinders University, Adelaide, South Australia, Australia; 7 Metro South and Integrated Nephrology and Transplant Services, Princess Alexandra Hospital, Woolloongabba, Queensland, Australia; 8 Metro North Hospital and Health Service, Royal Brisbane and Women’s Hospital, Herston, Queensland, Australia; Universidade Estadual Paulista Julio de Mesquita Filho, BRAZIL

## Abstract

**Rationale & objective:**

The prognostic significance of dialysis-dependent end-stage kidney disease on postoperative mortality is unclear. This study aims to estimate the odds of postoperative mortality in patients receiving chronic dialysis undergoing elective surgery compared to patients with normal kidney function, and to examine the influence of comorbidities on the excess mortality risk.

**Methods:**

A systematic search of studies published up to January 2020 was conducted using MEDLINE, EMBASE and CENTRAL databases. Eligible studies reported postoperative 30-day or in-hospital mortality in chronic dialysis patients compared to patients with normal kidney function undergoing elective surgery. Two investigators independently reviewed all abstracts and performed risk of bias assessments using the Newcastle-Ottawa Scale. Quality of evidence was summarised in accordance with GRADE methodology (grading of recommendations, assessment, development and evaluation). Relative mortality risk estimates were obtained using random effects meta-analysis. Heterogeneity was explored using meta-regression. (PROSPERO CRD42017076565)

**Results:**

Forty-nine studies involving 41, 822 chronic dialysis and 10, 476, 321 non-dialysis patients undergoing elective surgery were included. Patients on chronic dialysis had a greatly increased postoperative mortality odds compared to patients with normal kidney function. The excess risk ranged from OR 10.8 (95%CI 7.3–15.9) following orthopaedic surgery to OR 4.0 (95%CI 3.2–4.9) after vascular surgery. Adjustment for age and comorbidity attenuated the excess odds but remained higher for patients on chronic dialysis, irrespective of surgical discipline. Meta-regression analysis demonstrated an inverse linear relationship between excess mortality risk and study-level mean age (slope -0.06; P = 0.001) and diabetes prevalence (slope -0.02; p = 0.001).

**Conclusions:**

Patients on chronic dialysis have an increased odds for postoperative mortality following elective surgery across all surgical disciplines. This relationship is consistent among all studies, with the excess postoperative mortality attributable to end-stage kidney disease and chronic dialysis treatment may be lower among older patients with diabetes.

## Introduction

People with end-stage kidney disease (ESKD) on chronic dialysis experience high rates of hospitalisation, including the need for surgery, [1–3] and have been reported to have longer hospital stays and higher in-hospital mortality compared to patients with normal kidney function. [4, 5] It is unclear whether these heightened risks of postoperative complications are attributable to ESKD per se or the fact this group tend to be advanced in age and have a greater comorbid burden. [6, 7] In addition, published reports of postoperative outcomes in dialysis patients are limited by small studies, inconsistent adjustment for confounding factors, and lack of accounting for type and urgency of surgery. As a result, chronic dialysis patients are frequently deemed to be ‘high risk surgical candidates’; potentially affecting their access to surgical care [8].

Given that the number of people worldwide with ESKD requiring chronic dialysis is projected to double to 5.4 million by 2030, an increasing number of older, multimorbid chronic dialysis patients will contemplate elective surgery in the foreseeable future. [9] Therefore, ascertaining the excess postoperative risk for chronic dialysis patients would assist in planning elective surgery, mitigating risk and inform shared decision making between patients and clinicians in relation to potential benefits and harms of surgery.

The purposes of this study were to estimate the excess odds of fatal postoperative outcomes in dialysis-dependent patients compared to patients with normal kidney function, and to examine the influence of comorbidities on mortality.

## Methods

This systematic review adhered to the recommendations of the Meta-analysis Of Observational Studies in Epidemiology (MOOSE) and the Preferred Reporting Items for Systematic Reviews and Meta-Analysis (PRISMA) checklists, [10, 11] with a protocol registered in PROSPERO (CRD42017076565).

### Study eligibility criteria

All cohort studies that measured and reported postoperative mortality in adult (aged 18 years or older) chronic dialysis patients (haemodialysis and peritoneal dialysis) and in a control group of patients who had normal kidney function, as defined by serum creatinine less than 110μmol/l or absence of International Classification of Disease Coding (ICD) coding of chronic kidney disease and ESKD, were considered for inclusion. Non-dialysis dependant chronic kidney disease patients were excluded. All types of surgery requiring a general anaesthetic were considered, including general, orthopaedic, cardiac, vascular and urology/gynaecological surgery. Kidney transplant surgery and haemodialysis [HD]/peritoneal dialysis [PD] access surgery were excluded, as the former is performed in highly selected patients following rigorous cardiovascular evaluation, and the latter is considered minimally invasive surgery. [12, 13] More so, patients with normal kidney function would be ineligible for these surgeries. Since many studies reported data over a time period of several years, the data were assigned as close as possible to the median year in which the patients were recruited. Studies in which more than 20% of the procedures were emergent (defined as an acute illness leading to an emergency presentation or an unplanned admission requiring a surgical procedure) were excluded because emergent procedures have an inherently higher risk of perioperative complications. Studies reporting outcomes involving patient receiving continuous renal replacement therapy (CRRT) for acute kidney injury in the perioperative period were also excluded.

### Data sources and searches

MEDLINE, Embase and the Cochrane Controlled Register of trials (CENTRAL) were searched from inception to January 10th 2020, without language restriction using a combination of relevant keywords including *surgery*, *dialysis*, *postoperative*, *perioperative mortality* and their variants (S1, S2 and S3 Figs). Exploded MeSH terms for perioperative medicine and chronic dialysis patients were also used. Search terms were modified to correspond to the tree structure and descriptors of the two databases. Full-text articles obtained were hand searched for further references. Tangential electronic exploration using links to related texts was also performed for additional materials. Case-control studies, animal studies, opinion papers, case reports and editorials were excluded.

Database of Abstracts of Reviews of Effects (DARE), the Cochrane Database of Systematic Reviews (CDSR), National Institute for Health and Clinical Excellence (NICE) and the NIHR Health Technology Assessment (NIHR HTA) programme websites were all searched for existing reviews.

### Data extraction and quality assessment

Two authors (D.P. and A.N.) independently reviewed all titles and abstracts identified in the initial search to assess study eligibility, and any disagreements were resolved by a third reviewer (M.F.). Type of surgery, numbers of patient on chronic dialysis and patients with normal kidney function, summary statistics for baseline characteristics (including cardiovascular disease, peripheral vascular disease, diabetes mellitus, hypertension and smoking status), and frequency of postoperative outcomes in each group were extracted from full-text manuscripts of eligible studies using an electronic data extraction form. Additional data from corresponding authors was requested when required. The primary outcome was all-cause mortality, defined as either 30-day mortality or death within the same hospitalisation as the index surgery.

The methodological quality of each study was assessed in duplicate by 2 investigators (DP and AN) using the Newcastle-Ottawa Scale (NOS), which employs a star system to evaluate the selection of the study groups (0–4 stars), comparability of the groups (0–2 stars), and ascertainment of the outcome of interest (0–3 stars). [14]

Six reviewers (D.P., E.P, J.C., D.J., C.M., and M.F.) discussed overall quality of evidence and graded it on the basis outlined by Grading of Recommendations Assessment, Development and Evaluation (GRADE) working group. [15]

### Data synthesis and analysis

Comparative effect sizes were expressed as either relative risks, hazard ratios or odds ratios [OR] in the original articles. Unadjusted mortality ORs and 95% CIs were calculated using the number of events in each group. If no events occurred in one of the groups, then 0.5 was added to all cells in the 2 x 2 table to avoid computational difficulties with both the inverse variance and Mantel-Haenszel methods. In addition, if studies performed a multivariable analysis adjusted for age as a minimum, then the reported adjusted OR and 95% CI were recorded. Random effects meta-analysis was used to estimate the mean weighted effect within the different surgery types. Heterogeneity was assessed using both *I*^*2*^ to measure the percentage of variation in odds ratio estimate due to unobserved differences across studies. *I*^*2*^ tends to 100% as the number of patients included in a meta-analysis increases, and hence Tau^2^ is also provided to reflect absolute variation in odds estimates. [16, 17] Unadjusted and adjusted ORs for the primary outcome in dialysis versus patients with normal kidney function were pooled by surgical discipline. A pooled odds estimate across surgical disciplines was not calculated as postoperative risk is inherently related to the type of surgical procedure such that pooling across disciplines was not clinically relevant. The influence of comorbidities on the odds ratio estimate of postoperative mortality was assessed by a series of weighted univariable and multivariable random-effects meta-regression analyses. These analyses included two categories of predictor variables: study characteristics, including overall study quality (as per NOS), single versus multicentre cohorts, study continent (United States vs all other countries), median year of recruitment, surgery duration, single procedure studies versus composite procedures, and patient characteristics, including age and prevalence of diabetes mellitus or ischaemic heart disease among dialysis patients compared to patients with normal kidney function. The R^2^ index was used to quantify the proportion of variance in odds ratios explained by the covariates.

L’Abbé plots were generated to identify studies with divergent results as well as the study groups that were responsible for such differences. [18] Sensitivity analysis using meta-influence analysis was also performed to evaluate the influence of each study on the overall meta-analysis summary estimate and identify outlier studies that may have affected the validity of the conclusions. [19] Inter-rater reliability of study selection was assessed using Cohen’s kappa. A funnel plot and Egger’s test for funnel plot asymmetry were used to assess publication bias. Statistical analysis was performed with Stata 14.0 for Windows. Statistical significance was defined as a two-sided p-value <0.05.

## Results

### Description of included studies

**Fig 1:**
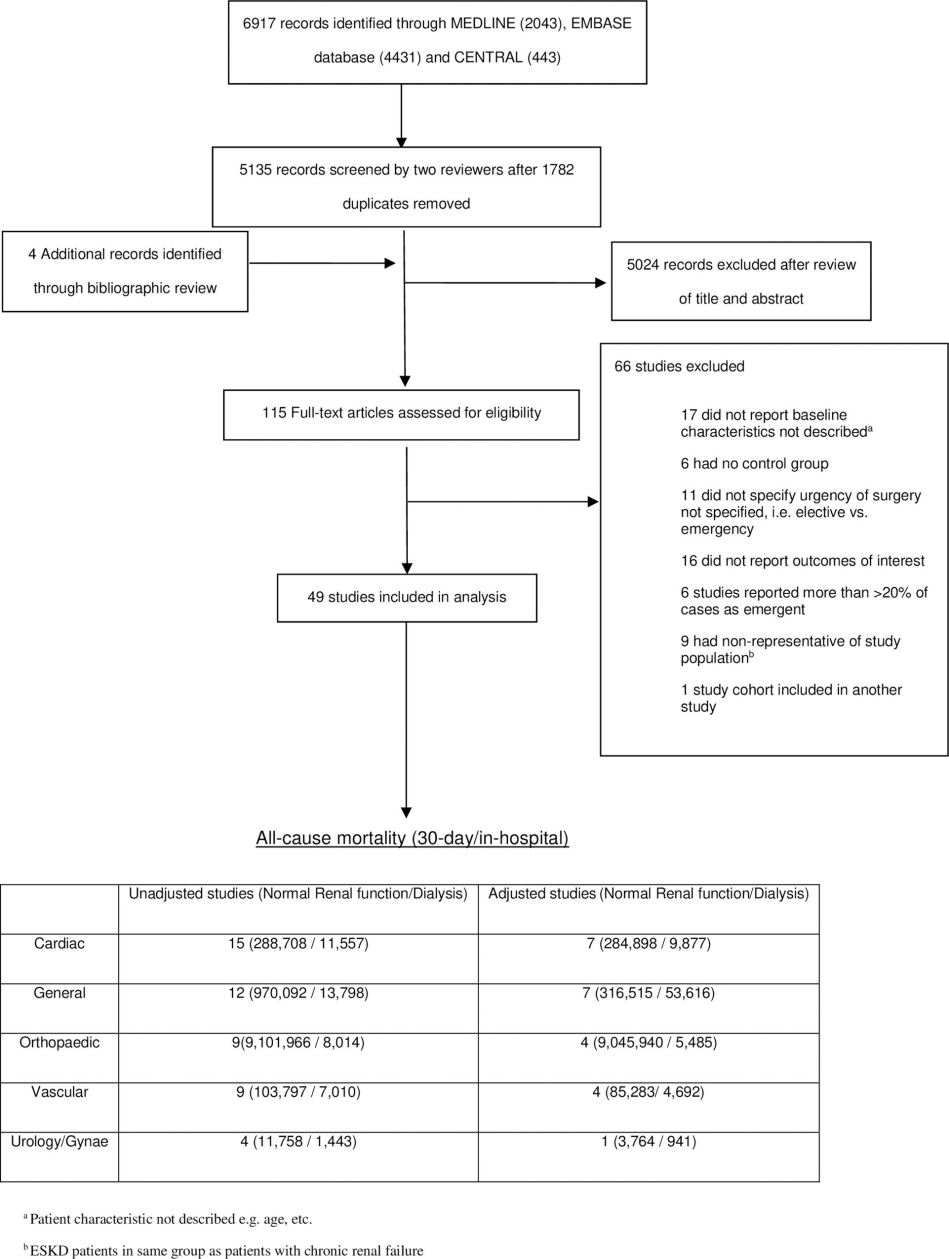
Study selection.

In total, 5135 abstracts were reviewed, from which 115 full-text articles were retrieved and evaluated (See Fig 1). 49 studies, involving 10,476,321 patients with normal kidney function and 41,822 chronic dialysis patients, satisfied the inclusion criteria. Table 1 lists the characteristics and designs of the 49 studies. The definition of chronic dialysis varied across studies, with 22 studies using registry-based definitions, seven using ICD coding and the remaining studies confirming chronic dialysis status by medical chart reviews. Twenty-one studies defined normal kidney function by serum creatinine. Non-emergent cardiac surgery was the most commonly reported type of surgery (15 studies, 31%), [20–34] followed by general surgery (12 studies, 25%), [4, 35–45] vascular surgery (9 studies, 18%), [41, 46–53] orthopaedic surgery (9 studies,18%) [54–61] and urologic/gynaecologic surgery (4 studies, 8%)(62–65). Twenty-two of the 49 studies assessed a single surgical procedure, [24–27, 29, 31–34, 39, 41, 43, 46, 53, 54, 56–58, 60–65] while the remaining 27 studies examined a combination of discipline-specific surgical interventions.

**Table 1:** Baseline characteristics of included studies.

Author	Country	Type of Surgery	Dialysis type	Total number of patients in study (n)		Mean age years (SD) Median age [IQR]		Ischaemic heart disease (%)		Diabetes (%)	
				Normal kidney function	Dialysis patients	Normal kidney function	Dialysis patients	Normal kidney function	Dialysis patients	Normal kidney function	Dialysis patients
**Cardiac Surgery**											
Al Sarraf [20], 2011	Ireland	Coronary artery bypass graft & Valve	Unspecified	3276	45	63.2(10.2)	62(12.3)	2763 (84.3)	36 (80)	560 (17.1)	10 (22.2)
Charytan [21], 2007	USA	Coronary artery bypass graft & Valve	Both	77323	635	66.1^‡^	62.9^‡^	14619 (18.9)	253 (39.8)	23208 (30.0)	367 (57.8)
Chikwe [22],2010	USA	Coronary artery bypass graft	Unspecified	2803	96	65.6(0.7)	63.3(10.7)	1398 (49.9)	52 (54.2)	1092 (38.9)	69 (71.9)
Cooper [23], 2006	USA	Coronary artery bypass graft & Valve	Unspecified	104880	7152	58.9^‡^	63^‡^	9439 (8.9)	1595 (22.3)	31464 (30)	4363 (61.0)
Fukushima [24], 2005	Japan	Coronary artery bypass graft	HD	451	20	67.2(8)	63.9(8)	451(100)	20 (100)	243 (53.9)	13 (65)
Griffin [34] 2019	USA	Coronary artery bypass and valve	HD	1416	35	62.6 (14.3)	58.8 (13.0)	1416 (100)	35 (100)	434 (30.7)	22 (62.9)
Kan [25], 2004	Taiwan	Coronary artery bypass graft	Both	69	23	63.8(11.2)	63.8(9.9)	23 (33.3)	23 (100)	34 (49.3)	15 (65.2)
Montgomery [45] 2019	USA	Bariatric surgery	Unspecified	417 403	1244	44.2 [35.7–53.3]	49.0 [41.6–55.8]	12497 (3.0)	199 (16.0)	109509 (26.2)	690 (55.5)
Murai [26], 2007	Japan	Coronary artery bypass graft	HD	60	39	67.2(7.9)	63.2(10.2)	60 (100)	39 (100)	NR	NR
Nakayama [27], 2003	Japan	Coronary artery bypass graft	Unspecified	794	84	65.9(9.4)	64.5(8.8)	794 (100)	84 (100)	245 (30.9)	35 (41.7)
Rahmanian [28], 2008	USA	Coronary artery bypass graft	Unspecified	6449	245	63.9(13.8)	61.3(13.2)	1548 (30.1)	81 (33.1)	1548 (24.0)	104 (42.4)
Raza [29], 2017	USA	Valve surgery	HD	199	144	58^‡^	55^‡^	NR	NR	NR	NR
Yamauchi [33], 2012	Japan	Coronary artery bypass graft	HD	18387	1300	68.7(9.4)	65.4(9.2)	NR	NR	8826 (48.0)	854 (65.7)
Wong [32] 2003	Canada	Coronary artery bypass graft	Unspecified	70	35	64(11)	64(11)	32 (45.7)	14 (40)	31 (44.3)	8 (22.9)
Vasileva [31] 2014	USA	Cardiac Valve surgery	Unspecified	85083	1480	63[52–72]	55[45–65]	NR	NR	10295 (12.1)	463 (31.3)
Thourani [30], 2012	USA	Coronary artery bypass graft and Valve	Haemodialysis	5084	224	61(14.8)	54(14.0)	798 (15.7)	46 (20.5)	933 (18.4)	87 (38.8)
**General Surgery**											
Andalib [35], 2016	USA	Bariatric procedures	Both	113677	234	44.7(11.6)	47.3(10.4)	154 (0.1)	1 (0.4)	30602 (26.9)	112 (47.9)
Barbas [36], 2014	USA	Hepatobiliary	Both	27275	101	62 [53–71]	60 [53–68]	2204 (8.1)	17 (16.8)	5431 (19.9)	48 (47.5)
Cheng [37], 2013	Taiwan	General	Both	8937	8937	65^‡^	65.5^‡^	3965 (44.4)	3930 (43.9)	4628 (51.8)	4589 (51.3)
Cloyd [38], 2014	USA	General	HD	24110	149	61.3(15.5)	62.3(13.4)	NR	NR	NR	NR
Ekici [39], 2009	Turkey	Laparoscopic Cholecystectomy	PD	33	11	45.6(11)	44.2(9.1)	NR	NR	NR	NR
Gajdos [4], 2013	USA	General surgery	Both	164094	1506	55.4(16.8)	59.6(15.1)	1590 (1.0)	100 (6.6)	26764 (16.3)	583 (38.7)
Hu [40], 2015	USA	Colorectal Surgery (General)	Unspecified	42138	265	70 [31–90]	67 [19–90]	NR	NR	131 (49.4)	7760 (18.4)
Marnique [44], 2017	Taiwan	Skin grafting for head and neck reconstruction	Unspecified	841	85	55.9(10.0)	56.3(10.5)	NR	NR	139(16.5)	52 (61.2)
Rao [41] 2014	USA	Cholecystectomy	Unspecified	80483	512	48.9(17.4)	59.8(14.9)	563 (0.7)	26 (0.1)	8853 (11.0)	235 (45.9)
Schneider [42], 2009	USA	General	Unspecified	108	54	59.8^‡^	59.3^‡^	NR	NR	NR	NR
Tam [43] 2015	USA	Ventral hernia (General)	Unspecified	90993	700	53.5(14.5)	56.7(13.5)	7097 (7.8)	160 (22.9)	12189 (13.4)	214 (30.6)
**Orthopaedic Surgery**											
Chikuda [54], 2012	Japan	Spinal surgery (Orthopaedic)	HD	50779	869	62.3(15.6)	64.3(8.2)	2597 (5.1)	126 (14.5)	6252 (12.3)	166 (19.1)
Cancienne [61] 2019	USA	Shoulder arthroplasty	Both	3675	1225	67.5^‡^	67.5^‡^	2570 (69.9)	881 (71.9)	2766 (75.3)	922 (75.3)
Chung [55], 2017	USA	Spinal surgery (Orthopaedic)	Unspecified	2522594	1834	59.9(14.7)	64.2(11.6)	48814 (1.9)	273 (14.9)	421178 (16.7)	907 (49.5)
Hickson [66] 2018	USA	Hip arthroplasty	Both	1508	377	78 [68–85]	77 [67–84]	NR	NR	648 (43.0)	162 (43.0)
Lizaur-Utrilla [56], 2016	Spain	Arthroplasty	HD	30	15	70.1[58–72]	69.3[56–72]	8 (26.7)	4 (26.7)	7 (23.3)	6 (40)
Ottesen [59], 2018	USA	Spinal surgery (Orthopaedic)	Unspecified	173311	467	57.0(5.5)	63(7.0)	NR	NR	NR	NR
Ottesen [60], 2018	USA	Total knee arthroplasty	Unspecified	163560	250	67.0(7.0)	68.0(7.0)	NR	NR	NR	NR
Ponumsamy [57], 2015	USA	Arthroplasty	Unspecified	6186475	2934	66.8(0.1)	66.7(0.6)	NR	NR	NR	NR
Yu [58] 2011	Taiwan	Spinal surgery (Orthopaedic)	HD	34	43	61.5(8.1)	62.5(8.1)	NR	NR	3 (8.8)	7 (16.3)
**Vascular surgery**											
Ambur [52] 2019	USA	Lower extremity bypass	Both	6101	550	68.2 (11.7)	66.4 (10.7)	188 (3.0)	42 (7.6)	2924 (47.9)	349 (63.5)
Balceniuk [53] 2019	USA	Distal lower extremity bypass	Both	12006	1014	68.4^‡^	67.3^‡^	209 (2.0)	29 (3.0)	5501 (57.0)	784 (81.0)
Gajdos [50], 2013	USA	Vascular procedures involving both peripheral arteries, carotid and aorta.	Both	34813	1409	70.3(10.8)	64.3(13.8)	7129 (20.5)	335 (23.8)	9029 (25.9)	726 (51.5)
Hibino [46], 2016	Japan	Abdominal aortic aneurysm repair (Vascular)	HD	679	21	65.7(13)	63.5(15.5)	NR	NR	79 (11.6)	9 (42.9)
Hickson [51], 2018	USA	Lower extremity amputation	Unspecified	5302	1166	68.0(11.0)	65.0(7.5)	NR	NR	3085 (58.2)	882 (75.6)
Lantis [47] 2001	USA	Infrainguinal bypass	Unspecified	481	60	69^‡^	63.0^‡^	241 (50.1)	38 (63.3)	212 (44.1)	50 (83.3)
Nicholas [48], 2000	USA	Infrainguinal bypass (Vascular)	Unspecified	46	57	72.3(9.1)	65.8(9.8)	35 (76.1)	55 (96.4)	29 (63.0)	46 (80.7)
O Hare [49] 2003	USA	Non-traumatic lower limb amputation	Unspecified	11051	1110	68^‡^	68^‡^	884 (8.0)	189 (17.0)	5747 (52.0)	799 (72.0)
Rao [67], 2017	USA	Infrainguinal bypass (Vascular)	Both	33318	1623	66.7(11.0)	66.0(10.7)	433(1.3)	73 (4.5)	12228 (36.7)	1034 (63.7)
**Urology/gynaecology surgery**											
Fornara [62], 1998	Germany	Laparoscopic Nephrectomy	HD	20	19	38 [24–70]	45 [20–73]	NR	NR	NR	NR
May [65] 2018	USA	Laprascopic nephrectomy	Both	7870	445	55	55	56 (0.7)	14 (3.1)	1637(20.8)	141 (31.7)
Schmitges [63], 2012	USA	Nephrectomy (Urologic/Gynaecologic)	Unspecified	3764	941	59(12.8)	59.1(12.7)	NR	NR	NR	NR
Yamashita [64] 2012	Japan	Nephrectomy (Urologic/Gynaecologic)	Both	104	38	61.9(14.1)	58.3(11.4)	NR	NR	NR	NR

‡ Standard deviation or interquartile range not provided

Thirty six studies did not report dialysis modality, eleven studies specifically examined haemodialysis patients only, [24, 26, 29, 30, 33, 34, 38, 40, 46, 54, 58, 62] and two studies reported on outcomes for patients on peritoneal dialysis separately. [39, 61] Only four studies recorded the cause of death. [25, 32, 46, 58]

Of the 49 studies, 19 reported findings from a single centre [20, 22, 24–30, 32, 39, 42, 46–48, 56, 58, 62, 64] and only three collected data prospectively. [20, 24, 58] Twenty-seven studies extracted information from existing data registries while the remaining extracted information from re-examined health records. Thirty-four studies were reported from North America, [4, 21–23, 28–31, 34–36, 38, 40–43, 45, 47–53, 55, 57, 59–61, 63, 65–67] 11 from Asia [24–27, 33, 37, 44, 46, 54, 58, 64] and 4 from Europe. [20, 39, 56, 62] Thirty four studies were published after 2010. [4, 20, 22, 29–31, 34–38, 40, 41, 43–46, 50–61, 63–67]

All 49 studies reported age and gender, but comorbidities were less consistently described, with 37 (76%) studies reporting the prevalence of diabetes mellitus, [4, 20–25, 27, 28, 30–37, 40, 41, 43, 45–56, 58, 61, 65–67] 31 (63%) reporting ischaemic heart disease(IHD), [4, 20–28, 30, 32, 34–37, 41, 43, 45, 47–50, 52–56, 61, 65, 67] 21 (43%) reporting smoking status, [23, 25, 30, 32–36, 40–42, 46–49, 51, 53, 67] and 15 studies (31%) reporting all three comorbidities. [32, 35, 36, 45, 47, 49, 52, 61, 65, 68–73]

### Meta-analyses: All-cause mortality

**Fig 2:**
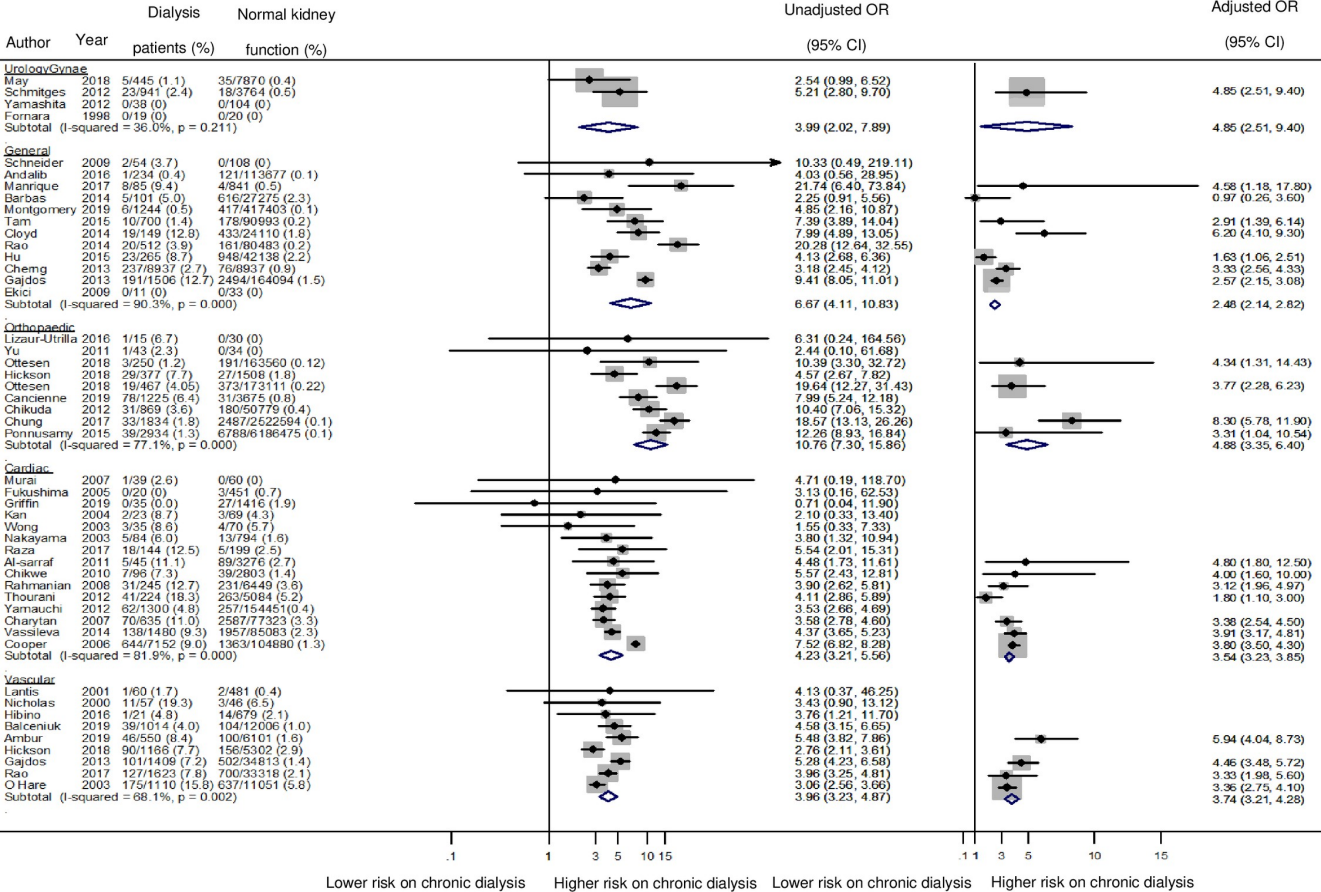
Odds of Postoperative mortality in patients on chronic dialysis (unadjusted and adjusted).

The incidence of in-hospital and/or 30-day mortality ranged from 0 to 19.3% in dialysis patients and from 0 to 7.7% in patients with normal kidney function, depending on surgical discipline and study (S1 Table). All studies consistently demonstrated an increased odds ratio of post-operative mortality in dialysis patients compared to patients with normal kidney function regardless of surgical discipline with the OR ranging from 3.96 (95%CI 3.23–4.87) to 10.76 (95%CI 7.30–15.86) (Fig 2).

The highest reported absolute median mortality rate was for chronic dialysis patients undergoing cardiac surgery (8.7%), followed by vascular surgery (7.8%). The largest odds ratio for postoperative mortality was observed for orthopaedic surgery (9 studies, 8014 dialysis patients, OR 10.76, 95% CI 7.30–15.86, I^2^ 77.1%, p for heterogeneity <0.001, Tau^2^ = 0.04, low certainty evidence), followed by general surgery (12 studies, 13 798 dialysis patients, OR 6.67, 95% CI 4.11–10.83, I^2^ 90.3%, p for heterogeneity <0.001, Tau^2^ = 0.56, low certainty evidence) and cardiac surgery (15 studies, 11 557 dialysis patients, OR 4.23, 95% CI 3.21–5.56, I^2^ 81.9, p for heterogeneity <0.001, Tau^2^ = 0.14, low certainty evidence). The lower bound of the 95%CI was greater than 1.0 in 73% of included studies.

Vascular surgery carried the lowest odds of postoperative mortality for chronic dialysis patients (9 studies, 7010 dialysis patients, OR 3.96, 95% CI 3.23–4.87 I^2^ = 68.1%, p for heterogeneity 0.003, Tau^2^ = 0.05, low certainty evidence).

Twenty-three studies examined risk of postoperative mortality after adjusting for covariates, with age as a minimum. Subgroup meta-analysis from the 23 studies showed that dialysis patients remained at higher risk of postoperative death compared to patients with normal kidney function (OR ranged from 2.48 to 4.88), but lower than the relative risk observed in studies reporting unadjusted analyses. Except for a single study by Barbas et al, which observed no significant differences in postoperative mortality odds between dialysis patients and patients with normal kidney function following pancreatic procedures (OR 0.97, 95% CI 0.26–3.60, p = 0.99, low certainty evidence), the ORs for death in all other individual studies showed a significantly greater mortality odds for dialysis patients compared to those with normal kidney function (Fig 2).

Pooled adjusted ORs by surgical discipline showed that orthopaedic surgery (4 studies, 5485 patients, OR 4.88, 95% CI 3.35–6.40, I^2^ 53.5%, p for heterogeneity = 0.091, Tau^2^ = 0.09, low certainty evidence) retained the highest mortality odds ratio for dialysis patients, followed by vascular surgery (4 studies, 4692 patients, OR 3.74, 95% CI 3.21–4.28, I^2^ 53.0%, p for heterogeneity = 0.094, Tau^2^ = 0.02, low certainty evidence) and cardiac surgery (7 studies, 9877 patients, OR 3.54, 95% CI 3.23–3.85, I^2^ 62.4%, p for heterogeneity = 0.014, Tau^2^ = 0.10, low certainty evidence).

Cause of death was poorly reported, thus formal analyses to evaluate cause-specific mortality were not performed.

### Explaining variation in relative risk estimates

**Fig 3:**
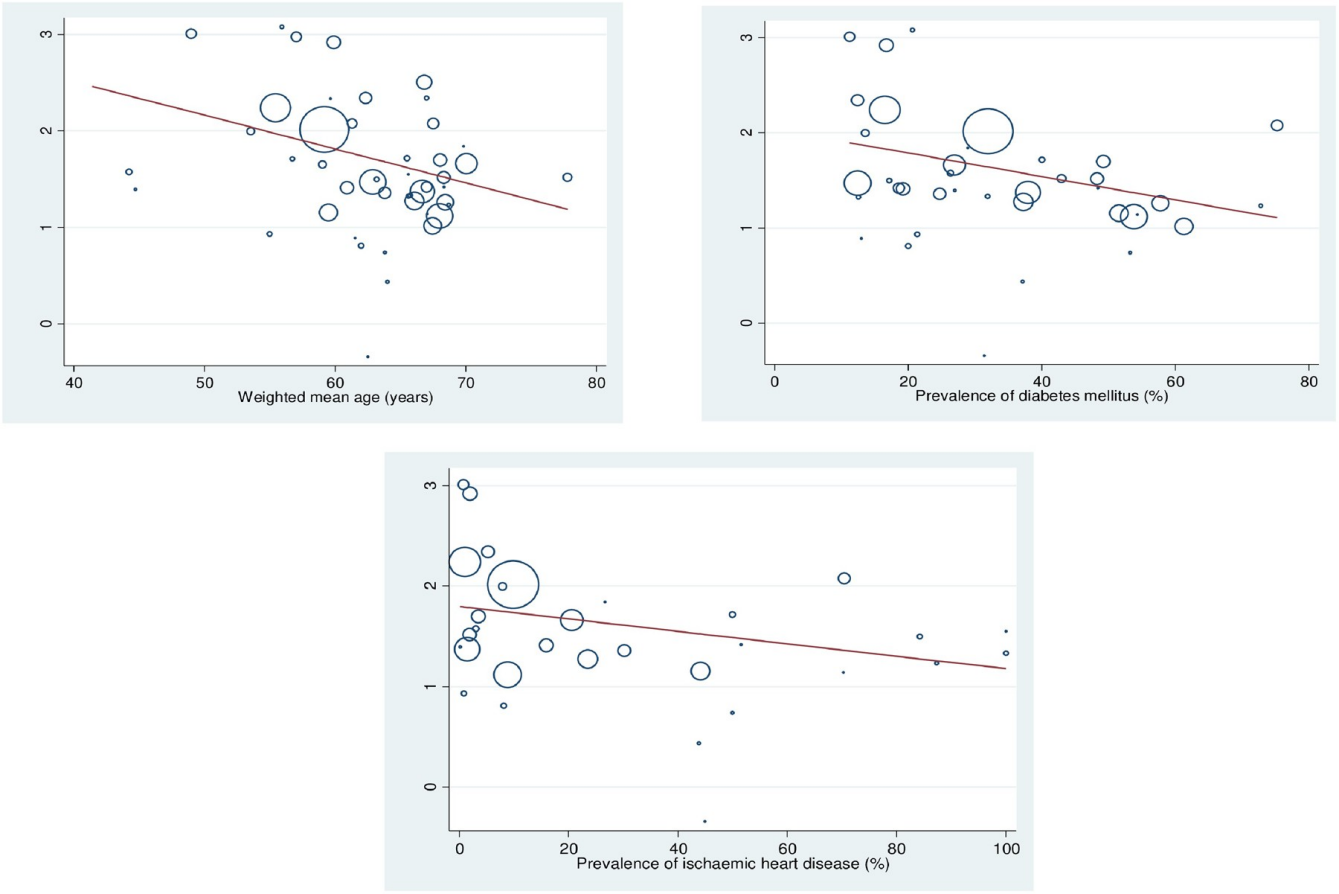
a-c, Meta-regression for postoperative mortality risk by mean age, prevalence of diabetes mellitus and ischaemic heart disease. a. Each circle represents a study; the circle size is representative of the weight of that study in the analysis. The relation between logarithmic mortality odds risk and the weighted mean age of dialysis and non-dialysis patients is significant (slope -0.04, 95% CI -0.06 –-0.01, *I2* 81.9%, adjusted R^2^ 18.5%, p = 0.018). b. Each circle represents a study; the circle size is representative of the weight of that study in the analysis. The relation between logarithmic mortality odds risk and the prevalence of diabetes mellitus of dialysis and non-dialysis patients is significant (slope -0.02, 95% CI -0.03 –-0.01, *I*^*2*^ 84.2%, adjusted R^2^ 19.6%, p = 0.022). c. Each circle represents a study; the circle size is representative of the weight of that study in the analysis. The relation between logarithmic mortality odds risk and the prevalence of ischemic heart disease of dialysis and non-dialysis patients is significant (slope -0.01, 95% CI -0.01–0.00, *I2* 88.7%, adjusted R^2^ 5.1%, p = 0.156).

A series of weighted univariable and multivariable random-effects meta-regression analyses were performed using study characteristics, including overall study quality (as per NOS), single versus multicentre cohorts, study continent, median year of recruitment, single procedure studies versus composite procedures, and cardiac versus non-cardiac surgery, none of which explained the variation in observed effect estimates. However, meta-regression with patient characteristics as predictor variables, including weighted mean age, prevalence of diabetes mellitus and ischaemic heart disease, did explain some of the variation in the observed mortality odds ratios. Univariable meta-regression of postoperative mortality odds ratio with weighted mean age of each study as a predictor variable showed that the logarithmic odds of postoperative mortality had an inverse linear relationship with mean age (slope -0.04 95%CI -0.06 – -0.01; p = 0.018; Fig 3A). A similar inverse relationship was found for diabetes (slope -0.02 95% CI -0.03–0.01; p = 0.022) but not ischaemic heart disease (slope -0.01 95% CI -0.01–0.00; p = 0.156, see Fig 3B and 3C, respectively). However, in multivariable meta-regression with all 3 factors, only diabetes mellitus was significant (slope -0.02 95% CI -0.03–0.00; p = 0.045).

### Risk of bias and certainty of evidence

As per the Newcastle-Ottawa Scale, reporting of outcomes was of good quality, but comparability of patient groups on the basis of analysis was poor in 26 (53%) studies due to the absence of multivariable adjustment for patient demographics and co-morbidities (S2 Table). [24–27, 29, 32, 33, 35, 39, 41, 42, 46–48, 51, 54, 56, 58, 62, 64]

L’Abbe plots and meta-influence analysis did not identify studies with outlying results or patient groups that may have been responsible for differences in estimates of relative mortality risk (S4 and S5 Figs).

There was no significant evidence of publication bias, as determined by funnel plot (S6 Fig) and Egger’s test (p = 0.328). Inter-rater variability between the two independent reviewers was strong (κ = 0.81).

The certainty in the quality of evidence used to estimate postoperative mortality odds was deemed to be low. The quality of evidence was downgraded due to serious concern with risk of bias and inconsistency in the magnitude of odds ratio estimates. The large and consistent direction of risk estimates improved the strength of evidence (S3 Table).

## Discussion

This systematic review and meta-analysis demonstrated an increase in postoperative mortality odds in patients with ESKD requiring dialysis compared to patients with normal kidney function following all types of elective surgery (OR range 4.0 (95%CI 3.2–4.9)– 10.8 (95%CI 7.3–15.9)). Sensitivity analyses including studies only reporting multivariable adjusted risk estimates reduced the odds (OR range 2.5 (95%CI 2.1–2.8)– 4.9 (95%CI 3.4–6.4)), highlighting the importance of judicious interpretation of odds ratios where comparator patients are not matched for important demographic and comorbid characteristics. Furthermore, our meta-regression analyses demonstrated that the excess odds for postoperative mortality attributable to receiving chronic dialysis was attenuated by increasing patient age and prevalence of diabetes, which are themselves established independent risk factors for post-operative mortality. [74] Having said that, a number of non-traditional risk factors associated with end-stage kidney disease including accelerated vascular calcification, mineral bone disease, anaemia, increased oxidative stress and impaired immunity, are all likely contributing factors [75–77].

Another potential reason for the observed elevated mortality odds, is the definition of dialysis dependent ESKD patients used in the studies. Major databases from which a number of studies were undertaken, including the American College of Surgeons National Surgical Quality Improvement Program (ACS NSQIP), Vascular Quality Improvement Program and The Society of Thoracic Surgeons Database (STS), do not record the presence of ESKD per se but rather the requirement of dialysis at the time of surgery to indicate the presence of ESKD without distinguishing between an acute kidney injury requiring dialysis and a patient with ESKD. Patients with severe acute kidney injury requiring dialysis tend to be sicker and have a substantially increased mortality odds at baseline, and therefore the inclusion of these patients may have potentially exaggerate the findings [78].

Nevertheless, patients considering kidney transplant surgery undergo rigorous cardiovascular assessment to identify occult coronary artery disease. However, no such recommendations exist to guide clinicians when contemplating elective surgery. In addition, studies comparing patient outcomes of surgical treatment to those of continued medical management are lacking.

In a prior meta-analysis of 31 cohort studies involving 125 930 patients with normal kidney function and 27 955 with non-dialysis-requiring chronic kidney disease, kidney dysfunction was identified as an important risk factor for post-operative death (OR 2.8; 95% CI 2.1–3.7), similar to that observed for diabetes, stroke and coronary artery disease. [6] Subsequent meta-regression demonstrated a graded relationship between declining glomerular filtration rate and post-operative mortality. The results of our meta-analysis with meta-regression support these findings and extend them by demonstrating that the odds of post-operative mortality remain elevated when patients transition to dialysis. Studies did not report cause of death thereby precluding further exploration of mechanisms of heightened risk. Indeed, adverse effects associated with ESKD and dialysis treatment itself on the cardiovascular and immune system may be important factors in the causal pathway. [79]

### Strengths and weaknesses

A comprehensive search strategy was used to identify published studies, ultimately pooling a large number of dialysis patients across all elective surgical types, allowing for greater generalisability to clinical care. Rigorous assessment of methodologic quality using a validated tool, robust ascertainment of patient-level outcomes, and use of meta-regression to explore heterogeneity and interactions are key strengths of this review. Nine studies did not provide statements on the adequacy of follow-up and eight did not explicitly state at the start of the study that the outcome of interest was not present.

Meta-regression identified that age and presence of diabetes attentuated the association between dialysis status and postoperative mortality, rather than specific attributes of the studies themselves. Furthermore, not all studies reporting adjusted results adjusted for the same covariates, so that adjustment by itself varied. Despite adjustment for potentital confounding variables, such as age, indication bias with residual confounding could not be excluded. Many of the studies did not report potentially important confounding variables, such as primary kidney disease, dialysis modality, vintage, and dialysis access type, residual kidney function or use of immunosuppression. The majority of studies occurred in North America thereby potentially limiting the generalisability of the review's findings. The definition of dialysis dependency varied across the studies, such that the possibility of misclassification of acute kidney injury requiring dialysis could not be excluded.

This review highlights the urgent need for future prospective studies to be more comprehensive in reporting patient baseline dialysis treatment characteristics (e.g. aetiology of kidney disease, dialysis modality, time on dialysis, access type, etc), procedural information and cause of death to allow for more informative analyses with adjustment for confounding, to help direct clinician’s perioperative risk assessment and efforts to minimise risk.

In conclusion, patients on chronic dialysis have a two- to fivefold increased odds of postoperative mortality following elective surgery. The magnitude of the excess odds attributable to dialysis dependent chronic kidney disease may be lower among older patients with diabetes.

## Supporting information

S1 Fig Search strategy to be used in EMBASE. (DOCX)

S2 Fig Search Strategy for MEDLINE. (DOCX)

S3 Fig Search strategy for CENTRAL. (DOCX)

S4 Fig L’Abbé plot for rates of all-cause mortality in the dialysis and control groups. (DOCX)

S5 Fig Meta-influence analysis for mortality. (DOCX)

S6 Fig Funnel plot. (DOCX)

S1 Table Summary characteristics of studies. (DOCX)

S2 Table Risk of bias assessment (based on Newcastle-Ottawa scale). (DOCX)

S3 Table GRADE summary of findings. (DOCX)

S1 Checklist (DOC)
