# Supplementary material for: Postoperative mortality in patients on chronic dialysis following elective surgery: A systematic review and meta-analysis
Source: PLoS One. 2020 Jun 26;15(6):e0234402. doi: 10.1371/journal.pone.0234402 (PMC7319352; doi:10.1371/journal.pone.0234402)
Supplement: S1 Fig — (DOCX) [file pone.0234402.s001.docx]

**Figure S1: Search strategy to be used in EMBASE**

1. 'dialysis':de,ti,ab
2. 'end-stage renal disease':de,ti,ab
3. 'renal replacement therapy':de,ti,ab
4. 'hemodialysis':de,ti,ab
5. 'peritoneal dialysis':de,ti,ab
6. 1 OR 2 OR 3 OR 4 OR 5
7. 'continuous renal replacement therapy'/exp
8. 'continuous renal replacement therapy'
9. 'acute kidney failure'/exp
10. 'acute kidney failure'
11. 'kidney transplantation'/exp
12. 'kidney transplantation'
13. 7 OR 8 OR 9 OR 10 OR 11 OR 12 OR 13
14. 6 NOT 13
15. surgery:de,ti,ab
16. 'postoperative complication'/mj
17. 'cardiovascular mortality'/exp
18. 'hospital mortality'/exp
19. 'surgical mortality'/exp
20. 'anastomosis dehiscence'/exp
21. 'anastomosis leakage'/exp
22. 'failed back surgery syndrome'/exp
23. 'gastric band erosion'/exp
24. 'vein graft disease'/exp
25. 'paravalvular leak'/exp
26. 'postoperative edema'/exp
27. 'postoperative hemorrhage'/exp
28. 'postoperative ileus'/exp
29. 'postoperative infection'/exp
30. 'postoperative inflammation'/exp
31. 'postoperative thrombosis'/exp
32. 'periprosthetic fracture'/exp
33. 'prosthetic valve dysfunction'/exp
34. 'surgical infection'/exp
35. 'surgical injury'/exp
36. 'heart infarction'/exp
37. 'cerebrovascular accident'/exp
38. 'hospital readmission'/exp
39. 'reoperation'/exp
40. 'blood transfusion'
41. 16 OR 17 OR 18 OR 19 OR 20 OR 21 OR 22 OR 23 OR 24 OR 25 OR 26 OR 27 OR 28 OR 29 OR 30 OR 31 OR 32 OR 33 OR 34 OR 35 OR 36 OR 37 OR 38 OR 39 OR 40
42. 14 AND 15 AND 41
