# Supplementary material for: Postoperative mortality in patients on chronic dialysis following elective surgery: A systematic review and meta-analysis
Source: PLoS One. 2020 Jun 26;15(6):e0234402. doi: 10.1371/journal.pone.0234402 (PMC7319352; doi:10.1371/journal.pone.0234402)
Supplement: S2 Fig — (DOCX) [file pone.0234402.s002.docx]

**Figure S2: Search Strategy for MEDLINE**

1. AB dialysis
2. TI dialysis
3. AB 'renal dialysis'
4. TI 'renal dialysis'
5. AB hemodialysis
6. TI hemodialysis
7. AB 'end stage renal disease'
8. AB 'end stage renal failure'
9. AB 'end stage kidney failure'
10. AB 'end stage kidney disease'
11. TI 'end stage renal disease'
12. TI 'end stage renal failure'
13. TI 'end stage kidney failure'
14. TI 'end stage kidney disease'
15. 1 OR 2 OR 3 OR 4 OR 5 OR 6 OR 7 OR 8 OR 9 OR 10 OR 11 OR 12 OR 13 OR 14
16. AB transplantation
17. TI transplantation
18. AB acute kidney injury
19. TI acute kidney injury
20. AB continous renal replacement therapy
21. TI continous renal replacement therapy
22. 16 OR 17 OR 18 OR 19 OR 20 OR 21 OR 22
23. 15 NOT 22
24. AB surgery
25. TI surgery
26. MM "Specialties, Surgical+"
27. 24 OR 25 OR 26
28. MM “Postoperative Complications”
29. MH "Cause of Death"
30. MH "Survival"
31. MH "Arrhythmias, Cardiac+"
32. MH "Death+"
33. MH "Hemorrhage+"
34. MH "Hypovolemia"
35. MH "Seroma"
36. MH "Serositis"
37. MH "Abscess"
38. MH "Cellulitis"
39. MH "Empyema"
40. MH "Systemic Inflammatory Response Syndrome+"
41. MH "Intraoperative Complications"
42. MH "Infarction"
43. MH "Fat Necrosis"
44. MH "Gangrene"
45. MH "Femur Head Necrosis"
46. MH "Anastomotic Leak"
47. MH "Graft Occlusion, Vascular"
48. MH "Incisional Hernia"
49. MH "Malignant Hyperthermia"
50. MH "Postcholecystectomy Syndrome"
51. MH "Postgastrectomy Syndromes"
52. MH "Postoperative Hemorrhage"
53. MH "Postpericardiotomy Syndrome"
54. MH "Prosthesis-Related Infections"
55. MH "Prosthesis Failure"
56. MH "Surgical Wound Dehiscence"
57. MH "Surgical Wound Infection"
58. MH "Respiratory Aspiration+"
59. MH "Multiple Organ Failure"
60. MH "Shock, Cardiogenic"
61. MH "Shock, Hemorrhagic"
62. MH "Ulcer"
63. MH "Arrhythmias, Cardiac"
64. MH "Death, Sudden, Cardiac"
65. MH "Out-of-Hospital Cardiac Arrest"
66. MH "Edema, Cardiac"
67. MH "Heart Failure, Diastolic"
68. MH "Heart Failure, Systolic"
69. MH "Acute Coronary Syndrome"
70. MH "Angina, Unstable+"
71. MH "Coronary Artery Disease"
72. MH "Coronary Occlusion"
73. MH "Anterior Wall Myocardial Infarction"
74. MH "Inferior Wall Myocardial Infarction"
75. MH "Non-ST Elevated Myocardial Infarction"
76. MH "Shock, Cardiogenic"
77. MH "ST Elevation Myocardial Infarction"
78. MH "Coronary Thrombosis"
79. MH "Postpericardiotomy Syndrome"
80. MH "Cerebral Infarction"
81. MH "Stroke, Lacunar"
82. MH "Brain Infarction"
83. MH "Pneumonia, Aspiration"
84. MH "Pneumonia, Bacterial"
85. MH "Pulmonary Atelectasis"
86. MH "Pulmonary Edema"
87. MH "Pulmonary Embolism"
88. MH "Length of Stay"
89. MH "Patient Readmission"
90. MH "Erythrocyte Transfusion"
91. MH "Blood Transfusion"
92. MH "Reoperation"
93. MH "Second-Look Surgery"
94. 28 OR 29 OR 30 OR 30 OR 31 OR 32 OR 33 OR 34 OR 35 OR 36 OR 37 OR 38 OR 39 40 OR 41 OR 42 OR 43 OR 44 OR 45 OR 46 OR 47 OR 48 OR 49 OR 50 OR 51 OR 52 OR 53 OR 54 OR 55 OR 56 OR 57 OR 58 OR 59 OR 60 OR 61 OR 62 OR 63 OR 64 OR 65 OR 66 OR 67 OR 68 OR 69 OR 70 OR 71 OR 72 OR 73 OR 74 OR 75 OR 76 OR 77 OR 78 OR 79 OR 80 OR 81 OR 82 OR 83 OR 84 OR 85 OR 86 OR 87 OR 88 OR 89 OR 90 OR 91 OR 92 OR 93
95. 23 AND 27 AND 94
