# Supplementary material for: Postoperative mortality in patients on chronic dialysis following elective surgery: A systematic review and meta-analysis
Source: PLoS One. 2020 Jun 26;15(6):e0234402. doi: 10.1371/journal.pone.0234402 (PMC7319352; doi:10.1371/journal.pone.0234402)
Supplement: S3 Fig — (DOCX) [file pone.0234402.s003.docx]

**Figure S3: Search strategy for CENTRAL**

1. #1 dialysis
2. #2 end stage kidney disease
3. #3 end stage renal disease
4. #4 hemodialysis
5. #5 peritoneal dialysis
6. #6 (dialysis):ti,ab,kw OR (end stage renal disease):ti,ab,kw OR (end stage kidney disease):ti,ab,kw OR (haemodialysis):ti,ab,kw OR (peritoneal dialysis):ti,ab,kw (Word variations have been searched)
7. #7 surgery
8. #8 perioperative outcomes
9. #9 perioperative mortality
10. #10 perioperative complications
11. #11 postoperative complications
12. #12 postoperative mortality
13. #13 #8 OR #9 OR #10 OR #11 OR #12
14. #14 #6 AND #7 AND #13
