# Supplementary material for: Postoperative mortality in patients on chronic dialysis following elective surgery: A systematic review and meta-analysis
Source: PLoS One. 2020 Jun 26;15(6):e0234402. doi: 10.1371/journal.pone.0234402 (PMC7319352; doi:10.1371/journal.pone.0234402)
Supplement: S4 Fig — (DOCX) [file pone.0234402.s004.docx]

**Figure S4: L’Abbé plot for rates of all-cause mortality in the dialysis and control groups**
