# Supplementary material for: Postoperative mortality in patients on chronic dialysis following elective surgery: A systematic review and meta-analysis
Source: PLoS One. 2020 Jun 26;15(6):e0234402. doi: 10.1371/journal.pone.0234402 (PMC7319352; doi:10.1371/journal.pone.0234402)
Supplement: S5 Fig — (DOCX) [file pone.0234402.s005.docx]

**Figure S5: Meta-influence analysis for mortality**

I^2^=87.4%

Estimate of between-study variance Tau-squared = 0.2012
