# Supplementary material for: Postoperative mortality in patients on chronic dialysis following elective surgery: A systematic review and meta-analysis
Source: PLoS One. 2020 Jun 26;15(6):e0234402. doi: 10.1371/journal.pone.0234402 (PMC7319352; doi:10.1371/journal.pone.0234402)
Supplement: S1 Table — (DOCX) [file pone.0234402.s007.docx]

**Table S1: Summary characteristics of studies**

|  |  | **Type of surgery** | | | | |
| --- | --- | --- | --- | --- | --- | --- |
|  |  | **Cardiac** | **General** | **Orthopaedic** | **Vascular** | **Urology/Gynae** |
| **Number of studies** |  | 15 | 12 | 9 | 9 | 4 |
| **Median Year of study recruitment** |  | 2002 | 2008 | 2007 | 2008 | 2005 |
| **Number of patients** | Dialysis | 11557 | 13798 | 8014 | 7010 | 1443 |
|  | Normal | 288708 | 970092 | 9,101,966 | 103797 | 11758 |
| **Median ages (years) [IQR]** | Dialysis | –63.0 [58.8 – 63.9] | 59.4 [52.3 – 59.9] | 67.5 [64.2 – 69.9] | 65.8 [64.0-66.4] | 56.7 [50.0-58.7] |
|  | Normal | 63.9 [62.6 – 66.1] | 55.7 [47.3 – 60.6] | 67.0 [61.5 – 72.3] | 68.2 [68.0-69.0] | 57.0[46.5-60.5] |
| **Median reported prevalence of Ischemic heart disease (%) [IQR]** | Dialysis | 58.5 [36.5-90.0] | 15.9 [5.1-22.9] | 22.2 [14.8-35.5] | 17.0 [4.5-63.3] | NR |
|  | Normal | 45.1 [26.7-77.1] | 3.0 [0.7-8.0] | 16.9 [5.1-26.7] | 8.0 [1.7-50.1] | NR |
| **Median reported prevalence of Diabetes Mellitus (%) [IQR]** | Dialysis | 57.8 [38.8-65.0] | 47.9 [45.9-51.3] | 41.5 [24.6-62.4] | 72.0 [63.5-77.3] | NR |
|  | Normal | 30.9 [24.0-44.3] | 18.4 [16.3-26.2] | 23.3 [14.0-59.1] | 45.8 [36.7-52.0] | NR |
| **Median reported mortality rates (%) [IQR]** | Dialysis | 8.7[4.8-11.1] | 3.8[1.0-9.1] | 3.4[1.8-6.4] | 7.8[4.0-14.3] | 0.6[0.0-1.8] |
|  | Normal | 2.3 [1.3-3.6] | 0.3[0.1-1.7] | 0.1[0.0-0.3] | 2.1[1.4-6.5] | 0.2[0.0-0.4] |

NR: Not reported
