# Supplementary material for: Postoperative mortality in patients on chronic dialysis following elective surgery: A systematic review and meta-analysis
Source: PLoS One. 2020 Jun 26;15(6):e0234402. doi: 10.1371/journal.pone.0234402 (PMC7319352; doi:10.1371/journal.pone.0234402)
Supplement: S2 Table — (DOCX) [file pone.0234402.s008.docx]

**Table S2: Risk of bias assessment (based on Newcastle-Ottawa scale)-**

| **First Author** | **Cohort Study Design** | **Dialysis Modality** | **Selection /🟑🟑🟑🟑** | **Comparability /🟑🟑** | **Outcomes /🟑🟑🟑** |
| --- | --- | --- | --- | --- | --- |
|  |  |  |  |  |  |
| Al Sarraf, 2011 | Retrospective | Unspecified | **🟑🟑🟑🟑** | **🟑🟑** | **🟑🟑🟑** |
| Ambur 2019 | Retrospective | Both | **🟑🟑🟑** | **🟑🟑** | **🟑🟑🟑** |
| Andalib, 2016 | Retrospective | Unspecified | **🟑🟑🟑🟑** | **-** | **🟑🟑🟑** |
| Balceniuk 2019 | Retrospective | Both | **🟑🟑🟑** | **🟑🟑** | **🟑🟑🟑** |
| Barbas, 2014 | Retrospective | Unspecified | **🟑🟑🟑** | **🟑🟑** | **🟑🟑🟑** |
| Cancienne 2019 | Retrospective | Both | **🟑🟑** | **-** | **🟑🟑** |
| Charytan, 2007 | Retrospective | Both | **🟑🟑🟑🟑** | **🟑🟑** | **🟑🟑🟑** |
| Cheng, 2013 | Retrospective | Both | **🟑🟑🟑** | **🟑🟑** | **🟑🟑🟑** |
| Chikuda, 2012 | Retrospective | Haemodialysis | **🟑🟑🟑** | **-** | **🟑🟑🟑** |
| Chikwe,2010 | Retrospective | Unspecified | **🟑🟑🟑🟑** | **🟑🟑** | **🟑🟑🟑** |
| Chung, 2017 | Retrospective | Unspecified | **🟑🟑🟑🟑** | **🟑🟑** | **🟑🟑🟑** |
| Cloyd, 2014 | Retrospective | Haemodialysis | **🟑🟑** | **🟑🟑** | **🟑🟑🟑** |
| Cooper, 2006 | Retrospective | Unspecified | **🟑🟑🟑🟑** | **🟑🟑** | **🟑🟑🟑** |
| Ekici, 2009 | Retrospective | Peritoneal Dialysis | **🟑🟑🟑🟑** | **-** | **🟑🟑🟑** |
| Fornara, 1998 | Retrospective | Haemodialysis | **🟑🟑🟑** | **-** | **🟑🟑🟑** |
| Fukushima, 2005 | Prospective | Haemodialysis | **🟑🟑🟑** | **-** | **🟑🟑🟑** |
| Gajdos, 2013 | Retrospective | Both | **🟑🟑🟑🟑** | **🟑🟑** | **🟑🟑🟑** |
| Gajdos, 2013 | Retrospective | Both | **🟑🟑🟑🟑** | **🟑🟑** | **🟑🟑🟑** |
| Griffin 2019 | Retrospective | Haemodialysis | **🟑🟑🟑🟑** | **-** | **🟑🟑🟑** |
| Hibino, 2016 | Retrospective | Haemodialysis | **🟑🟑🟑🟑** | **-** | **🟑🟑🟑** |
| Hickson, 2018 | Retrospective | Haemodialysis | **🟑🟑🟑🟑** | **-** | **🟑🟑🟑** |
| Hickson 2018 | Retrospective | Both | **🟑🟑🟑🟑** | **-** | **🟑🟑🟑** |
| Inoue 2018 | Retrospective | Both | **🟑🟑🟑** | **-** | **🟑🟑🟑** |
| Hu, 2015 | Retrospective | Unspecified | **🟑🟑🟑🟑** | **🟑🟑** | **🟑🟑🟑** |
| Kan, 2004 | Retrospective | Both | **🟑🟑🟑🟑** | **-** | **🟑🟑🟑** |
| Lantis 2001 | Retrospective | Unspecified | **🟑🟑🟑** | **-** | **🟑🟑🟑** |
| Lin 2019 | Retrospective | Unspecified | **🟑🟑🟑** | **🟑🟑** | **🟑🟑** |
| Lizaur-Utril, 2016 | Retrospective | Unspecified | **🟑🟑🟑🟑** | **-** | **🟑🟑🟑** |
| Marique, 2017 | Retrospective | Unspecified | **🟑🟑🟑🟑** | **🟑🟑** | **🟑🟑🟑** |
| May 2018 | Retrospective | Both | **🟑🟑🟑** | **-** | **🟑🟑🟑** |
| Montgomery 2019 | Retrospective | Unspecified | **🟑🟑🟑🟑** | **-** | **🟑🟑🟑** |
| Murai, 2007 | Retrospective | Haemodialysis | **🟑🟑🟑🟑** | **-** | **🟑🟑** |
| Nakayama, 2003 | Retrospective | Unspecified | **🟑🟑🟑🟑** | **-** | **🟑🟑🟑** |
| Nicholas, 2000 | Retrospective | Unspecified | **🟑🟑** | **-** | **🟑🟑** |
| O Hare | Retrospective | Unspecified | **🟑🟑🟑🟑** | **🟑🟑** | **🟑🟑🟑** |
| Ottesen, 2018 | Retrospective | Unspecified | **🟑🟑🟑** | **🟑🟑** | **🟑🟑🟑** |
| Ottesen, 2018 | Retrospective | Unspecified | **🟑🟑🟑** | **🟑🟑** | **🟑🟑🟑** |
| Ponumsamy, 2015 | Retrospective | Unspecified | **🟑🟑🟑** | **🟑🟑** | **🟑🟑🟑** |
| Rahmanian, 2008 | Retrospective | Unspecified | **🟑🟑🟑** | **🟑🟑** | **🟑🟑🟑** |
| Rao, 2017 | Retrospective | Unspecified | **🟑🟑🟑** | **🟑🟑** | **🟑🟑🟑** |
| Rao 2014 | Retrospective | Unspecified | **🟑🟑🟑** | **-** | **🟑🟑** |
| Raza, 2017 | Retrospective | Haemodialysis | **🟑🟑🟑** | **-** | **🟑🟑** |
| Schmitges, 2012 | Retrospective | Unspecified | **🟑🟑🟑** | **🟑🟑** | **🟑🟑** |
| Schneider, 2009 | Retrospective | Unspecified | **🟑🟑🟑** | **-** | **🟑🟑** |
| Tam 2015 | Retrospective | Unspecified | **🟑🟑🟑🟑** | **🟑🟑** | **🟑🟑** |
| Thourani, 2012 | Retrospective | Haemodialysis | **🟑🟑🟑🟑** | **🟑🟑** | **🟑🟑🟑** |
| Vasileva 2014 | Retrospective | Unspecified | **🟑🟑🟑🟑** | **🟑🟑** | **🟑🟑🟑** |
| Wong 2003 | Retrospective | Unspecified | **🟑🟑🟑🟑** | **-** | **🟑🟑🟑** |
| Yamashita 2012 | Retrospective | Both | **🟑🟑🟑🟑** | **-** | **🟑🟑🟑** |
| Yamauchi, 2012 | Retrospective | Haemodialysis | **🟑🟑🟑🟑** | **-** | **🟑🟑🟑** |
| Yu 2011 | Prospective | Haemodialysis | **🟑🟑🟑🟑** | **-** | **🟑🟑🟑** |

**Selection criteria (out of 4 stars):** Representation of exposed cohort, selection of non-exposed group, ascertainment of exposure, demonstration that outcome of interest was not present at start of study

**Comparability (out of 2 stars):** Comparability of cohorts by the design or analysis

**Outcomes (out of 3 stars):** Assessment of outcomes, Follow-up duration and adequacy of follow-up of cohorts
