# Supplementary material for: Postoperative mortality in patients on chronic dialysis following elective surgery: A systematic review and meta-analysis
Source: PLoS One. 2020 Jun 26;15(6):e0234402. doi: 10.1371/journal.pone.0234402 (PMC7319352; doi:10.1371/journal.pone.0234402)
Supplement: S3 Table — (DOCX) [file pone.0234402.s009.docx]

**Table S3: GRADE Summary of findings table**

| **Outcomes (Number of Studies)** | **Events in chronic dialysis patients** | **Events in normal kidney function** | **Unadjusted odds ratio (95% CI)** | **Certainty of evidence (GRADE)** | **Rationale** | | | | | |
| --- | --- | --- | --- | --- | --- | --- | --- | --- | --- | --- |
|  |  |  |  |  | **Risk of bias** | **Inconsistency** | **Indirectness** | **Imprecision** | **Publication bias** | **Strong association** |
| Cardiac surgery (15) | 1,027  /11,557 | 6,841  /288,708 | 4.23 (3.21-5.56) | Low ^a,d,f^ | ↓ | ↓ | – | – | – | ↑ |
| General surgery (12) | 522  /13,798 | 5,448  /970,092 | 6.67(4.11-10.83) | Low ^a,b,d,e^ | ↓ | ↓ | – | – | – | ↑↑ |
| Orthopaedic surgery (9) | 234  /8,014 | 10,077  /9,101,966 | 10.76(7.30-15.86) | Low ^a,b,d,e^ | ↓ | ↓ | – | – | * | ↑↑ |
| Vascular surgery (9) | 594  /7,010 | 2,244  /103,797 | 3.96(3.23-4.87) | Low ^a,b,d,f^ | ↓ | – | – | – | * | ↑ |
| Urology/Gynaecology (4) | 28  /1,443 | 53  /11,758 | 3.99 (2.02-7.89) | Very low ^a,b,c,d,f^ | ↓ | ↓ | – | ↓↓ | * | ↑ |

^a^ Certainty of evidence downgraded due to concerns of poor comparability between study cohorts (as assessed using Newcastle-Ottawa Scale)

^b^ Certainty of evidence downgraded for inconsistency due to heterogeneity that was not completely explained using meta-regression (study and patient characteristics)

^c^ Certainty of evidence downgraded for imprecision, due to few events and wide confidence intervals.

*^, d^ Publication bias not judged to assess certainty of evidence due to small study numbers (<10)

^e^ Certainty of evidence upgraded due to large (>5 fold) risk estimate

^f^ Certainty of evidence upgraded due to large (>2 fold) risk estimate
